# Taxonomic review of the *Cataglyphis livida* complex (Hymenoptera, Formicidae), with a description of a new species from Iran

**DOI:** 10.3897/zookeys.1010.58348

**Published:** 2021-01-13

**Authors:** Sebastian Salata, Haniyeh Kiyani, Kambiz Minaei, Lech Borowiec

**Affiliations:** 1 Department of Biodiversity and Evolutionary Taxonomy, University of Wrocław, Przybyszewskiego 65, 51–148, Wrocław, Poland California Academy of Sciences San Francisco United States of America; 2 California Academy of Sciences, 55 Music Concourse Dr, San Francisco, 94118, CA, USA University of Wrocław Wrocław Poland; 3 Department of Plant Protection, Faculty of Agriculture, Shiraz University, Shiraz, Iran Shiraz University Shiraz Iran

**Keywords:** *
Cataglyphis
*, eastern Mediterranean, key to species, south-western Iran, taxonomy

## Abstract

*Cataglyphis fici***sp. nov.**, a member of the *Cataglyphis livida* complex, is described from the Estahban county of south-western Iran. The species is characterized by small body size and yellow to yellowish red body coloration with distinctly infuscated legs. Additionally, the taxonomic status of all known members of the *Cataglyphis livida* complex is updated. *Cataglyphis lutea* Pisarski, 1967, **stat. rev.** is raised to the species level and *Cataglyphis viaticoides* (André, 1881) is proposed as a senior synonym of *Cataglyphis livida bulgarica* Atanassov, 1982, **syn. nov.** and *Cataglyphis albicans mixtus* (Forel, 1895), **syn. nov.** Finally, a provisional key to members of the *livida* complex is provided.

## Introduction

The ant genus *Cataglyphis* Foerster, 1850 currently includes 112 valid species and subspecies (Bolton 2020) distributed mostly in the semideserts and deserts of the Palearctic Region and the sub-Saharan area, India, and Pakistan. *Cataglyphis* species are among the most common ants occurring in arid and open, Mediterranean-type habitats of North Africa, the Arabian Peninsula, and Central Asia. Less frequently, *Cataglyphis* was recorded from the high altitude, mountain steppes, or forest steppes (Agosti 1990; Radchenko 1997; Brown 2000). Members of the genus are thermophilous, nest in ground and feed mainly on dead insects and other invertebrates. They are characterized by a strictly diurnal activity and are recognized for their superior navigating abilities (Lenoir et al. 2009; Wehner 2020).

The very first worldwide revision of the genus (Santschi 1929) is outdated. The only modern and comprehensive revision of *Cataglyphis* was presented by Agosti (1990) who provided, among others, its redefined species group division. Unfortunately, most of the groups recognized by Agosti remain unrevised and consist of taxa of uncertain status. On the regional level *Cataglyphis* was revised in the following countries: Armenia (Arakelian 1994), Bulgaria (Atanassov and Dlussky 1992), China (Chang and He 2002), Iraq (Pisarski 1965), Kingdom of Saudi Arabia (Collingwood and Agosti 1996), Morocco (Cagniant 2009), Portugal (Collingwood and Prince 1998), and Turkmenistan (Dlussky et al. 1992). There are also available revisions or checklists covering the former European U.S.S.R. (Arnol’di and Dlussky 1978), Iberian Peninsula (Collingwood 1978), Asia (Radchenko 1997, 1998), Central and North Europe (Seifert 2018), and Arabian Peninsula (Collingwood et al. 2011; Sharaf et al. 2015).

Recent publications, presenting descriptions of new species and changes in taxonomic statuses of these already described, proved that the diversity of *Cataglyphis* is underestimated and requires further studies (Radchenko and Paknia 2010; Amor and Ortega 2014; Sharaf et al. 2015; Ionescu and Eyer 2016; Salata and Borowiec 2018). Iran, due to its location and predominance of open and arid habitats, hosts one of the highest numbers of *Cataglyphis* species. So far, there are 35 *Cataglyphis* taxa recorded for this country, but some records need confirmation or correction (Paknia et al. 2008, 2009; Moradloo et al. 2015; Janicki et al. 2016; Rad et al. 2018; Khalili-Moghadam et al. 2021).

*Cataglyphis fici*, a new species collected in Fars Province of Iran, is a member of the *Cataglyphis albicans* species group sensu Agosti (1990). Its members are characterized by small body size (WL < 3.5 mm), monomorphic workers and colonies lacking distinct major or soldier castes, nodiform petiole with angled dorsal outline and short peduncle, subtly microsculptured and shiny body, and uniformly yellow to black mesosoma. Within the *albicans* species group, *C. fici* is most similar to species listed within the *livida* complex. Agosti (1990) characterized this complex based on uniform, yellow body coloration and included there three species: *C. argentata* (Radoszkowsky), *C. livida* (André), and *C. viaticoides* (André), four subspecies: *C. albicans aurata* Menozzi, *C. albicans fezzanensis* Bernard, *C. albicans mixtus* (Forel), and *C. livida lutea* Pisarski, and four quadrinominal unavailable names: *C. livida lutea agnata* Santschi, *C. livida lutea ambigua* Santschi, *C. livida lutea arabica* (Emery), and *C. livida luteaarenaria* Finzi. Members of the *livida* complex are distributed from Morocco to Indus river including the Arabian Peninsula, and inhabit semi-deserts, deserts, and rocky open areas such as dry hills or coastal cliffs.

The work presented here is a contribution to studies on members of the *C. livida* complex. We list an updated synopsis of members of this complex and provide a provisional key to their identification. Additionally, we describe *Cataglyphis fici* sp. nov., a new member of the *C. livida* complex, based on material recently collected from Iran.

## Materials and methods

Investigated specimens were collected in fig orchards located in Estahban city, Fars Province, Iran and are part of the material gathered for a scientific project conducted by the second author. The city is placed 1730 m a.s.l and is characterized by a dry climate, with a yearly precipitation amount of 224 millimeters and summer temperatures frequently exceeding 25.0 °C.

The dominant method was direct sampling (hand collecting). Individual specimens were collected on the ground and preserved in 75% EtOH. Photographs were taken using a Nikon SMZ 1500 stereomicroscope, Nikon D5200 photo camera, and Helicon Focus software. All given label data are in the original spelling, presented in square brackets; a vertical bar (|) separates data on different rows and double vertical bars (||) separate labels. Type specimens’ photographs are available online on AntWeb (www.AntWeb.org) and are accessible using the unique CASENT identifying specimen code.

Examined specimens are housed in the following collections:

**MNHW** Museum of Natural History, University of Wrocław, Poland, in temporary deposit by Department of Biodiversity and Evolutionary Taxonomy, University of Wrocław, Poland;

**MHNG**Muséum d’Historie Naturelle, Genève, Switzerland.

Measurements:

**HL** head length; measured in a straight line from mid-point of anterior clypeal margin to mid-point of posterior margin in full-face view;

**HW** head width; measured in full-face view at the center of the eyes;

**SL** scape length; maximum straight-line length of scape excluding the basal condylar bulb;

**PW** pronotum width; maximum width of pronotum in dorsal view;

**PRL** propodeum length; measured in lateral view, from metanotal groove to posterior-most point of propodeum;

**PRW** propodeum width; maximum width of propodeum in dorsal view;

**PTH** petiole height; the chord of ventral petiolar profile at node level is the reference line perpendicular to which the maximum height of petiole is measured, measured in lateral view;

**PTW** petiole width; maximum width of the petiolar node in lateral view;

**WL** Weber’s length; measured as diagonal length from the anterior end of the neck shield to the posterior margin of the propodeal lobe;

**HFL** hind femur length; measured on dorsal side from trochanter to apex of femur.

All measurements are given in mm.

Ratios

**CI** cephalic index, HL/HW;

**SI** scape index, SL/HL;

**PI** petiole index, PTH/PTW;

**FI** femur index, HFL/WL.

### Synopsis of species of the *Cataglyphis livida* complex

*Cataglyphis arenaria* Finzi, 1940

*Cataglyphis argentata* (Radoszkowsky, 1876)^1^

*Cataglyphis aurata* Menozzi, 1932

*Cataglyphis fici* sp. nov.

*Cataglyphis livida* (André, 1881)

*Cataglyphis lutea* Pisarski, 1967, stat. rev.

*Cataglyphis viaticoides* (André, 1881)

= *Cataglyphis livida bulgarica* Atanassov, 1982, syn. nov.

= *Cataglyphis albicans mixtus* (Forel, 1895), syn. nov.

## Taxonomy

### Diagnosis of workers of the *Cataglyphis livida* complex

Small body size (WL < 3.0 mm); colonies with monomorphic workers, lacking distinct major or soldier castes; petiole nodiform with angled dorsal outline and short peduncle; body subtly microsculptured and shiny; body uniformly yellow to red (never brown to black) or bicolored with entirely to partially black gaster.

Distribution: from Morocco to Asia Minor and the Middle East, in semideserts, deserts and rocky open areas such as dry hills or coastal cliffs.

Note 1. Agosti (1990), as the first one, noticed that the data from labels of type specimens of *C. viaticoides* did not correspond with the original description of the species. This problem was later investigated and clarified by Bračko et al. (2016). Based on evidence gathered and discussed by the authors, the definition of the *livida* complex proposed by Agosti (1990) was modified to accommodate *C. viaticoides*.

Note 2. Based on its description, *C. albicans fezzanensis* Bernard is characterized by the presence of polymorphic worker caste, and additional study on the type specimen indicated that its body sculpture is stronger and less shiny than in other members of the *livida* complex. Based on this data, we decided not to list this species as a member of this complex.

### Provisional key to the *Cataglyphis livida* complex

**Table:** 

1	At least mid and hind legs infuscated. Iran	***C. fici***
–	Legs in the same coloration as mesosoma	**2**
2	Head and mesosoma uniformly yellowish red to reddish yellow, gaster entirely or mostly black, Balkans to Asia Minor	***C. viaticoides***
–	Gaster in the same coloration as the rest of body (yellow to red) or its apex slightly infuscated	**3**
3	Mesosoma and head without layer of silvery hair, northeastern Mediterranean to Middle East	***C. lutea***
–	At least mesosoma with a layer of silvery hair, Morocco to Asia Minor	**4**
4	Mesosoma and posterior head with thick layer of silvery hair, North Africa	***C. arenaria* , *C. argentata* , *C. aurata*** ^2^
–	A layer of silvery hair limited to mesosoma, Asia Minor	***C. livida***

### Review of species

#### Cataglyphis fici sp. nov.

Taxon classificationAnimaliaHymenopteraFormicidae

AC0D5BEC-1837-5858-AC5B-EDF206B11E0A

http://zoobank.org/81413366-4DA0-44D9-BF48-3ACD395E86F4

**Figures 1, 2.**
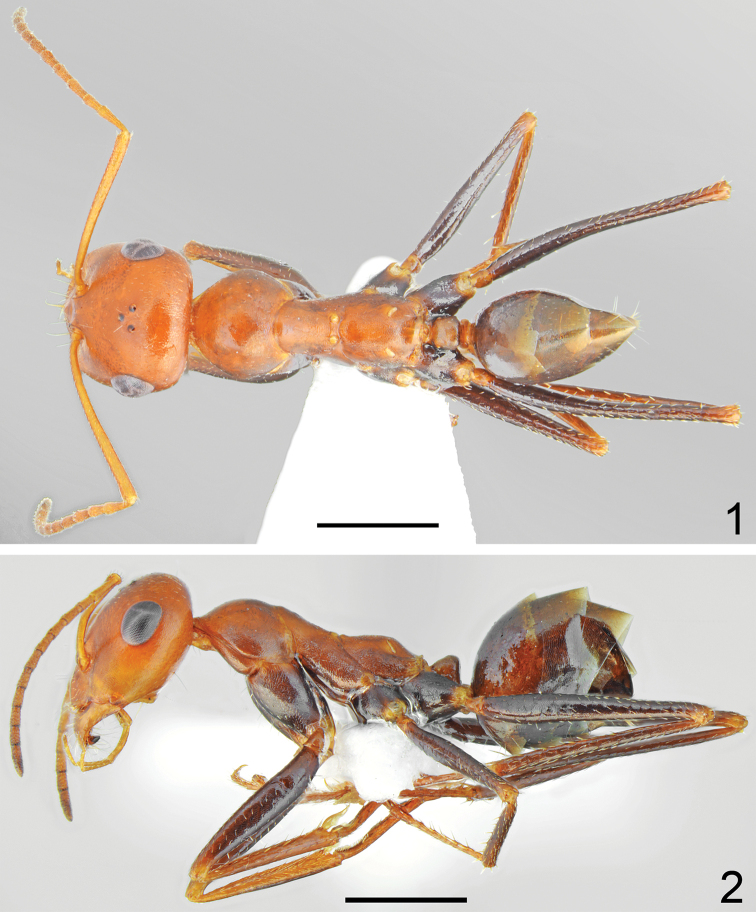
Holotype worker of *Cataglyphis fici* sp. nov. **1** dorsal **2** lateral. Scale bars: 1 mm.

**Figures 3, 4.**
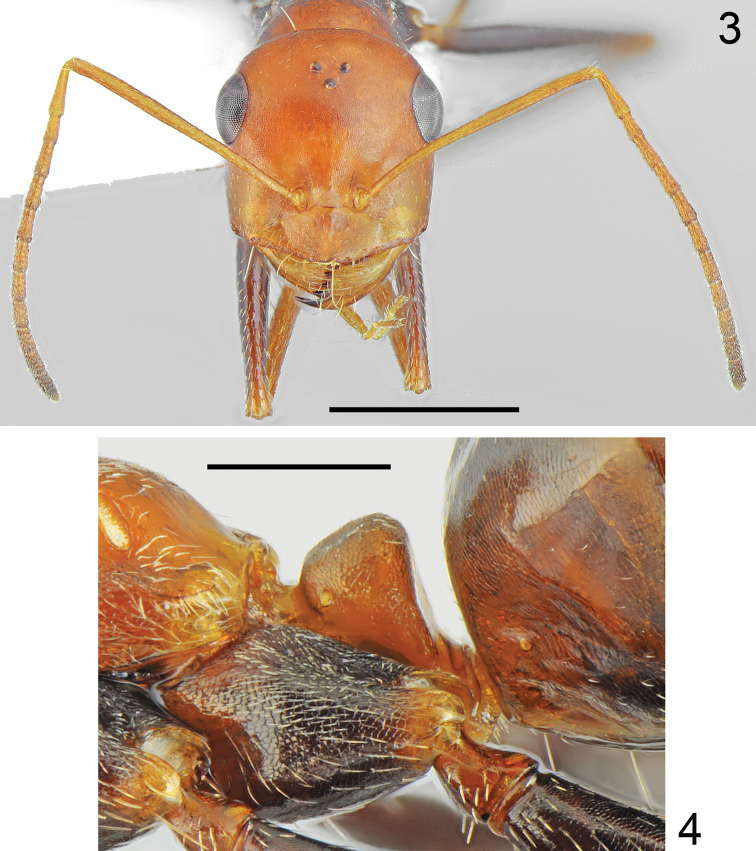
Holotype worker of *Cataglyphis fici* sp. nov. **3** head **4** petiole Scale bars: 0.5 mm (**4**), 1 mm (**3**).

**Figures 5, 6.**
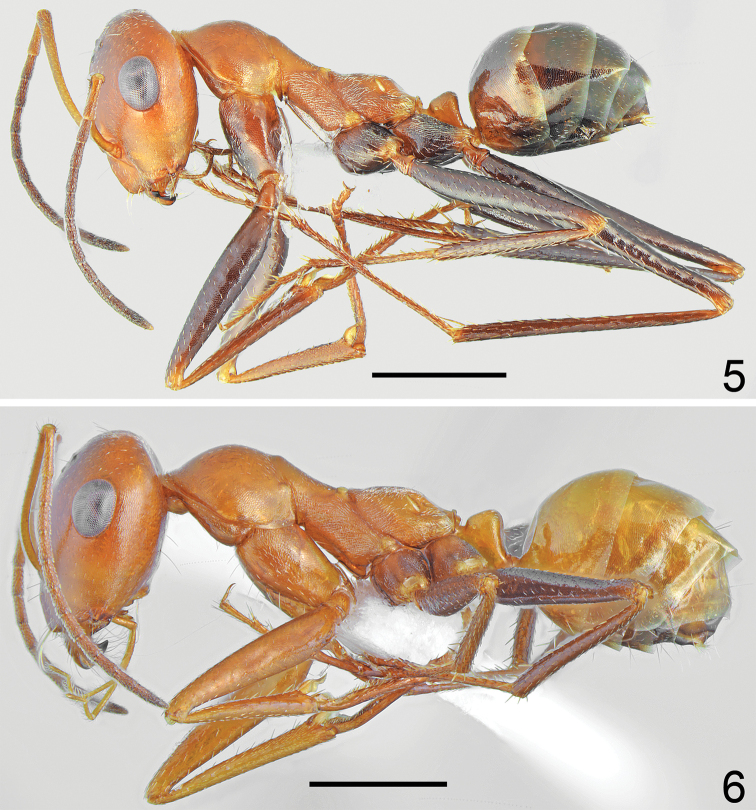
Paratype workers of *Cataglyphis fici* sp. nov. **5** the darkest form **6** the palest form Scale bar: 1 mm.

Figs 1–2 , 3, 4 , 5–6

##### Type material.

***Holotype***: Iran •worker, Fars, Estahban, 29.1331/54.389, 1730 m a.s.l., 16 Aug. 2019, H. Kiyani leg., LBC-IR00179, CASENT6006519 (MNHW); ***paratypes***: 5 workers, the same data as holotype, CASENT6006520–CASENT6006524 (MNHW, MHNG); ***paratype***: worker: the same data as holotype except LBC-IR00180, CASENT6006525 (MNHW); ***paratype***: worker, the same data as holotype except 6 Sep. 2018 and LBC-IR00182, CASENT6006526 (MNHW).

***Holotype worker labels***: Iran, Fars, 1730 m | Estahban | 29.1331 / 54.389 | 16 VIII 2019, H. Kiyani || Collection L. Borowiec | Formicidae | LBC-IR00179 || CASENT6006519.

##### Diagnosis.

*Cataglyphis fici* is a member of the *Cataglyphis albicans* group and can be separated from all species clustered in the *cinnamomea* and *fortis* complexes and most of members of the *albicans* complex by yellow to yellowish red body coloration; while other species have body completely brown to black. From bicolored species of the *albicans* complex it differs in smaller body size (WL < 3 mm) and presence of infuscated to yellowish brown mid and hind legs. However, *C. fici* is most similar to species included in the *C. livida* complex and can be easily separated based on at least mid and hind legs partly to mostly infuscated to yellowish brown. In contrast, all remaining members of the *C. livida* complex have legs uniformly colored and always in the same shade as mesosoma and head. Additionally, *C. fici* differs from *C. lutea*, *C. arenaria*, *C. argentata*, *C. aurata*, and *C. livida* in gaster darker than head and mesosoma sometimes infuscated.

##### Description.

Worker (n = 8): ***Measurements.***HL: 1.405 (1.29–1.54); HW: 1.325 (1.23–1.43); SL: 1.483 (1.36–1.59); PW: 0.897 (0.84–0.97); PRL: 0.737 (0.68–0.79); PRW: 0.595 (0.56–0.65); PTH: 0.383 (0.32–0.654); PTW: 0.360 (0.34–0.38); WL: 1.933 (1.81–2.09); HFL: 2.038 (1.83–2.24); CI: 1.060 (1.038–1.077); SI: 1.056 (1.032–1.072); PI: 1.065 (0.889–1.158); FI: 1.054 (1.036–1.111). ***Color.*** Head, mesosoma and petiolar scale from yellow to yellowish red, in the darkest specimens sides of mesonotum and propodeum indistinctly infuscated, gaster in the palest specimens mostly yellow with infuscated two apical sternites (Fig. 6), in dark specimens yellowish at base then gradually infuscated apically, with dark brown apical tergites and sternites (Fig. 5). Forelegs in the palest specimens completely yellow, mid and hind legs with brown femora and tibiae and yellowish tarsi, in dark specimens all legs at least partly infuscated. Usually fore coxa mostly brown with reddish spots of diffused borders laterally, fore femora mostly brown with yellowish apices, fore tibiae yellowish brown and fore tarsi yellowish; mid and hind femora dark brown, tibiae yellowish brown to brown, tarsi yellowish to yellowish brown (Figs 2, 5). Antennal scape yellow, funicles in the palest specimens slightly darker than scape, yellowish basally and yellowish brown apically, in dark specimens only first segment of funiculus yellowish, remaining segments gradually yellowish brown to dark brown. ***Head.*** Subrectangular, approximately 1.05 × as long as wide, sides below eyes almost parallel, above eyes gently convex, occipital margin convex (Fig. 3). Anterior margin of the clypeus convex, with small median emargination, clypeal anterior margin with a row of short, white setae and additional six long, white setae, the longest as long as 0.7 × length of clypeus. Whole surface of clypeus densely microreticulate with shiny background, covered with very sparse and short, adpressed hairs. Eyes large, oval, approximately 1.4 × as long as wide. Frontal carinae short, not extending beyond frontal lobes, interocular area without shiny line or carina and with a pair of long white setae. Antennal fossa shallow, microreticulate with shiny background. Whole head surface finely microreticulate with shiny background, occipital part of the head and are behind eyes with reduced sculpture and shinier, covered with extremely sparse, indistinct, adpressed hairs. Ocellar region with a pair of moderately long white setae, occipital part of head with 2–6 long, white erect setae, underside only with a pair of long, white setae close to lateral margin of head. Antennal scape moderately long, in frontal view almost straight, approximately 1.1 × as long as length of the head; thin, in apex only slightly and gradually widened; its base without tooth. Funiculus long, first funicle segment elongated, approximately 0.8 × as long as segments II and III combined, and 1.7 × as long as segment II (Fig. 3). Surface of scape densely microsculptured; shiny to indistinctly opalescent; covered with strong, moderately dense, decumbent setae. Mandibles rounded, only in basal part smooth and shiny, apical ¾ with deep grooves, surface shiny with several long yellow setae, masticatory margin with four4 large teeth. ***Mesosoma.*** Long, 2.1 × as long as wide; metanotal groove shallow (Fig. 2). Pronotum convex on sides (Fig. 1). In lateral view promesonotum slightly arched in profile; propodeum positioned lower than promesonotum, moderately convex in lateral view; propodeal spiracle strongly elongated and slit-shaped, approximately 4.2 × as long as wide (Fig. 2). Whole mesosoma opalescent, with dense, fine microreticulation and shiny background; covered with extremely sparse and short adpressed microsetae, on sides of pro- and mesonotum appears almost hairless, only anterior part of pronotum, posterior angles of mesonotum and propodeum with sparse setosity. Pro- and mesonotum without erect setae, propodeum without erect setae or in its posterior part with one or two short, white, erect setae. ***Scale*.** In form of a short cuneiform node, in lateral view almost trapezoidal with very short peduncle. Anterior face close to base distinctly convex, posterior face slightly concave, top of scale in lateral view obtusely rounded, without erect setae (Fig. 4). In anterior and posterior view top margin of scale without emargination. Surface of petiole distinctly microreticulate and shiny. ***Gaster.*** With fine transverse microreticulation and striation and very shiny background. Whole surface of gaster with hardly visible, extremely short, sparse, adpressed microsetae, tergites I and II without erect setae, tergites III and IV with a pair of long white setae centrally, each gastral sternite with 2–4 long, white, erect setae. ***Legs.*** Dorsal and lateral surface of femora and tibiae covered with sparse, white adpressed setae. Ventral surface of femora and tibiae with rows of elongate, white, erect spiniform setae.

##### Biology.

Little known, workers were collected on the ground in fig orchard at altitude 1730 m.

##### Etymology.

The species name *fici* is a noun in the genitive case named after the generic name of the fig tree, *Ficus* sp., the dominant plant in the type locality of this ant species.

#### Cataglyphis arenaria

Taxon classificationAnimaliaHymenopteraFormicidae

Finzi, 1940

09B4BC47-F887-556E-80ED-7C41BDC64654

Cataglyphis (Cataglyphis) albicans var. arenaria Finzi, 1940: 164 [first available use of Myrmecocystus albicans lividusarenaria Forel, 1909: 384].
Status as species: Collingwood and Agosti (1996): 378.
Syntype worker, Biskra, Algeria (MHNG) [Syntype worker images examined, AntWeb, CASENT0911101, photographs by Alexandra Westrich, available on AntWeb.org].

##### Diagnosis.

Whole body yellow, only gaster sometimes with indistinctly infuscated apex; mesosoma, posterior part of the head and coxae covered with a layer of silvery hair.

##### Distribution.

North Africa region, from Mauritania to Jordan. Based on photographs available on AntWeb specimens from Arabian Peninsula probably refer to *C. lutea*.

##### Note.

*Cataglyphis arenaria* was separated from *C. livida* and *C. viaticoides* based on the presence of a thick layer of silvery hair on mesosoma and posterior part of the head. Two years after the original description of *C. arenaria*, Karavaiev (1911) described Myrmecocystus albicans ssp. lividus var. aurata, which was later validated by Menozzi (1932) as trinominal. The latter species also was separated from *C. livida* based on presence of silvery hair on its body. Probably, Karavaiev was unaware of the existence of *C. arenaria* during his work on *C. aurata*. Study of type specimens and descriptions of both *C. arenaria* and *C. aurata* did not provide any characters useful in separating these two species. Thus, we conclude that both taxa could be conspecific (see also note in *Cataglyphis argentata* (Radoszkowsky, 1876) and *C. aurata* Menozzi, 1932). However, this hypothesis requires verification based on larger material collected from the whole area of their distribution, supported with studies on male genitalia, and genetic analyses.

#### Cataglyphis argentata

Taxon classificationAnimaliaHymenopteraFormicidae

(Radoszkowsky, 1876)

F839526F-1035-56F9-8321-0D87A7CC7B0B

Camponotus argentata Radoszkowsky, 1876: 140.
Cataglyphis argentata : Dalla Torre (1893): 217.
Type specimens. Unavailable.

##### Diagnosis.

Whole body yellow, only gaster sometimes with indistinctly infuscated apex; mesosoma, body covered with a layer of silvery hair.

##### Distribution.

Egypt.

##### Note.

Type specimens of this species are considered lost and, as suggested by Agosti (1990), due to ambiguous description of this species, its assignation to the *livida* complex is tentative. The silvery hair mentioned in the description can suggest an affiliation of *C. argentata* with the *bombycina* or *laevior* complexes. If *C. argentata* is a member of the *livida* complex, then its description could indicate that it is probably conspecific with two other North African taxa: *C. arenaria* and *C. aurata*. If this assumption is correct, then the name *C. argentata* has priority over *C. arenaria* and *C. aurata*.

#### Cataglyphis aurata

Taxon classificationAnimaliaHymenopteraFormicidae

Menozzi, 1932

2D9CB448-01B9-534C-984D-941829C99ECB

Cataglyphis (Cataglyphis) albicans aurata Menozzi, 1932: 95 [first available use of Myrmecocystus albicans ssp. lividus var. auratus Karavaiev, 1911: 10].
Syntype worker, Assuan, Egypt (MHNG) [syntype worker images examined, AntWeb, CASENT0911100, photos by Zach Lieberman, available on AntWeb.org].

##### Diagnosis.

Whole body yellow, only gaster sometimes with indistinctly infuscated apex; mesosoma, posterior head and coxa covered with a layer of silvery hair.

##### Distribution.

North Africa. Probably records from Asia Minor refer to *Cataglyphis lutea*.

##### Note.

*Cataglyphis aurata* was separated from *C. livida* based on the presence of a thick layer of silvery hair on its body. Probably, Karavaiev, during his work on *C. aurata*, was unaware of the existence of *C. arenaria*, another species described from the North African region characterized by the same feature. Study on type specimens and descriptions of both *C. arenaria* and *C. aurata* did not provide any characters useful separating these two species. Thus, we conclude that they could be conspecific. However, this hypothesis requires verification based on larger material collected from the whole area of their distribution, supported with studies on male genitalia, and genetic analyses. See also note in *C. argentata*.

#### Cataglyphis livida

Taxon classificationAnimaliaHymenopteraFormicidae

(André, 1881)

8E60E5FB-DFC1-5866-8BC9-84F3337BFF5E

Myrmecocystus albicans var. lividus André, 1881: 58.
Status as species: Arnol’di (1964): 1810.
Syntype workers, Jaffa, Israel (MHNG) [syntype workers images examined, AntWeb, CASENT0911099 and CASENT0912207, photographs by Zach Lieberman and Will Ericson, available on AntWeb.org].

##### Diagnosis.

Whole body yellow, only gaster sometimes with indistinctly infuscated apex; mesosoma and coxa covered with a layer of silvery hair.

##### Distribution.

Unknown. Due to mislabeling of type specimens of *C. livida* and *C. viaticoides*, both species were wrongly interpreted, and most of their historic records require verification. Based on available material, we can confirm its presence in Egypt, coastal parts of Israel, Syria, and Antalya Province in Turkey.

##### Note.

A study on type specimens of *C. livida* revealed that this species could be easily separated from most members of the *livida* complex based on the presence of a layer of silvery hair on propodeum and katepisternum, and lack of these on posterior head. Lack of comment on this feature in the original description combined with mislabeling of type specimens (see Bračko et al. 2016) caused confusion, leading to the long-lasting misinterpretation of *C. livida*.

#### Cataglyphis lutea

Taxon classificationAnimaliaHymenopteraFormicidae

Pisarski, 1967 stat. rev.

385202E0-C7B4-5AE2-84D5-4FD13B4F4CDF

Cataglyphis livida subsp. lutea Pisarski, 1967: 418 [first available use of Myrmecocystus albicans viaticoideslutea Emery, 1906: 53].
Junior synonym of Cataglyphis livida: Radchenko, 1997: 428.
Syntype worker, Shiraz, Iran (MSNG) [Syntype worker images examined, AntWeb, CASENT0905718, photographs by Will Ericson, available on AntWeb.org].

##### Diagnosis.

Whole body yellow, only gaster sometimes with indistinctly infuscated apex; body never with a layer of silvery hair.

##### Distribution.

Species known from Arabian Peninsula east to Afghanistan.

##### Note.

*Cataglyphis lutea* was described from Shiraz, Fars Province in Iran as an unavailable quadrinominal name (Emery 1906), later validated by Pisarski (1967) as a subspecies of *C. livida*, and finally considered as its junior synonym (Radchenko 1997). A study on type specimen revealed that *C. lutea* distinctly differs from *C. livida* in lack of a layer of silvery hair on mesosoma, and its distribution does not overlap with confirmed records of *C. livida*. Thus, we decided to raise it to the species status. AntCat resources indicated that, except type locality, *C. lutea* is also known from Aran va Bidgol, Maranjab, Iran (CDA000106) and Saudi Arabia (CASENT0906455).

#### Cataglyphis viaticoides

Taxon classificationAnimaliaHymenopteraFormicidae

(André, 1881)

018559C6-0A0A-5B6D-AABD-3F8D398CD221

Myrmecocystus albicans var. viaticoides André, 1881: 57.
Syntype worker, Beirut, Lebanon (MNHN) [syntype worker images examined, AntWeb, CASENT0912236, photographs by Zach Lieberman, available on AntWeb.org].
= Cataglyphis livida bulgarica subsp. bulgarica Atanassov, 1982: 213, syn. nov.
Type specimens unavailable.
= Myrmecocystus albicans var. mixtus Forel, 1895: 229, syn. nov.
Syntype worker, Edirne, Turkey (MHNG) [syntype worker images examined, AntWeb, CASENT0911104, photographs by Zach Lieberman, available on AntWeb.org].

##### Diagnosis.

Head and mesosoma uniformly yellowish red to reddish yellow, gaster entirely or mostly dark; thin layer of silvery hair limited to propodeum.

##### Distribution.

Balkans and Asia Minor.

##### Note.

Radchenko (1997), based on confusion related to the type labels of *C. livida* and *C. viaticoides* (see Bračko et al. 2016), considered *C. livida bulgarica* and *C. albicans mixtus* as junior synonyms of *C. livida*. Results presented by Bračko et al. (2016) clarified that the only member of the *livida* complex with entirely or mostly black gaster is *C. viaticoides* and thus *Cataglyphis livida bulgarica* Atanassov, 1982 and *Cataglyphis albicans mixtus* (Forel, 1895) should be considered as its junior synonyms.

## Supplementary Material

XML Treatment for Cataglyphis fici

XML Treatment for Cataglyphis arenaria

XML Treatment for Cataglyphis argentata

XML Treatment for Cataglyphis aurata

XML Treatment for Cataglyphis livida

XML Treatment for Cataglyphis lutea

XML Treatment for Cataglyphis viaticoides

## Footnotes

Due to ambiguous description of this species it is tentatively assigned to the livida complex.
